# Tyr66 acts as a conformational switch in the closed-to-open transition of the SHP-2 N-SH2-domain phosphotyrosine-peptide binding cleft

**DOI:** 10.1186/1472-6807-7-14

**Published:** 2007-03-22

**Authors:** Olgun Guvench, Cheng-Kui Qu, Alexander D MacKerell

**Affiliations:** 1 Department of Pharmaceutical Sciences, University of Maryland School of Pharmacy, 20 Penn St., HSF II-629, Baltimore, MD 21201, USA; 2 Department of Medicine, Division of Hematology/Oncology, Case Comprehensive Cancer Center, Case Western Reserve University, 10900 Euclid Ave., Cleveland, OH 44106, USA

## Abstract

**Background:**

The N-terminal SH2 domain (N-SH2) of the non-receptor tyrosine phosphatase SHP-2 is involved both in localization of SHP-2 by recognition of phosphotyrosine (pY) peptides and self-inhibition of SHP-2 phosphatase activity through the formation of a protein – protein interface with the phosphatase domain. Mutations that disrupt this interface break the coupling between pY-peptide binding cleft conformation and self-inhibition, thereby increasing both SHP-2 phosphatase activity and pY-peptide binding affinity, and are associated with the congenital condition Noonan syndrome and various pediatric leukemias. To better characterize the molecular process involved in N-SH2 pY-dependent binding, we have applied explicit-solvent molecular dynamics simulations to study the closed-to-open transition of the N-SH2 pY-peptide binding cleft.

**Results:**

The existence of stable conformations in the left-handed helical and the extended regions of Tyr66 φ/ψ space prevent rapid interconversion of the backbone and create a conformational switch such that Tyr66 in a left-handed helical backbone conformation results in an open cleft and in an extended backbone conformation results in a closed cleft. The stable conformations arise from deep, well-localized free-energy minima in the left-handed helical and extended regions of the Tyr66 φ/ψ map. Changing the Tyr66 backbone conformation from extended to left-handed helical induces a closed-to-open transition in the cleft, and the reverse change in backbone conformation induces the reverse, open-to-closed transition. In the open-cleft state, weak solvent-exposed interactions involving the sidechains of Tyr66, Asp40, Lys55, and Gln57 serve to anchor the Tyr66 sidechain to the surface of the protein and away from the binding cleft entrance, thereby facilitating pY-peptide access to the binding cleft.

**Conclusion:**

The simulations point to a regulatory role for Tyr66 and surrounding residues in SHP-2 function: mutations at Tyr66, Asp40, Lys55, and/or Gln57 are predicted to break the switching mechanism and negatively impact pY-peptide binding. This in turn would interfere with cellular localization and the coupled SHP-2 phosphatase activity. The structurally well-defined binding cleft conformations resulting from the switch-like transition suggest the possibility of applying structure-based methods to develop inhibitors of N-SH2 pY-peptide binding to serve as research tools for signal transduction and precursors to therapeutics for SHP-2-related diseases.

## Background

The ubiquitously expressed vertebrate non-transmembrane protein tyrosine phosphatase SHP-2 takes part in intracellular signal transduction induced by a variety of environmental cues and plays an important role in diverse cellular processes [1-3]. The SHP-2 protein consists of 593 residues, with the first 213 residues comprising two SRC homology 2 domains (SH2) and the remainder a protein tyrosine phosphatase domain (PTP) and the C-terminal tail. The 2.0 Å X-ray crystal structure of SHP-2 [4] reveals that the PTP catalytic site is blocked by the formation of an intramolecular protein – protein interface between PTP and the N-terminal SH2 domain (N-SH2), thereby providing a structural explanation for the low baseline SHP-2 tyrosine phosphatase activity [5,6]. In addition to self-inhibiting catalysis, N-SH2, like the second (C-terminal) SH2 domain (C-SH2), has the capacity to selectively bind phosphotyrosine (pY) peptides of a particular sequence [7,8]. Thus, SHP-2 can be recruited to different regions of the cell via the interaction of its N-SH2 or C-SH2 domains with particular pY-peptides localized in these different regions.

**Figure 1:**
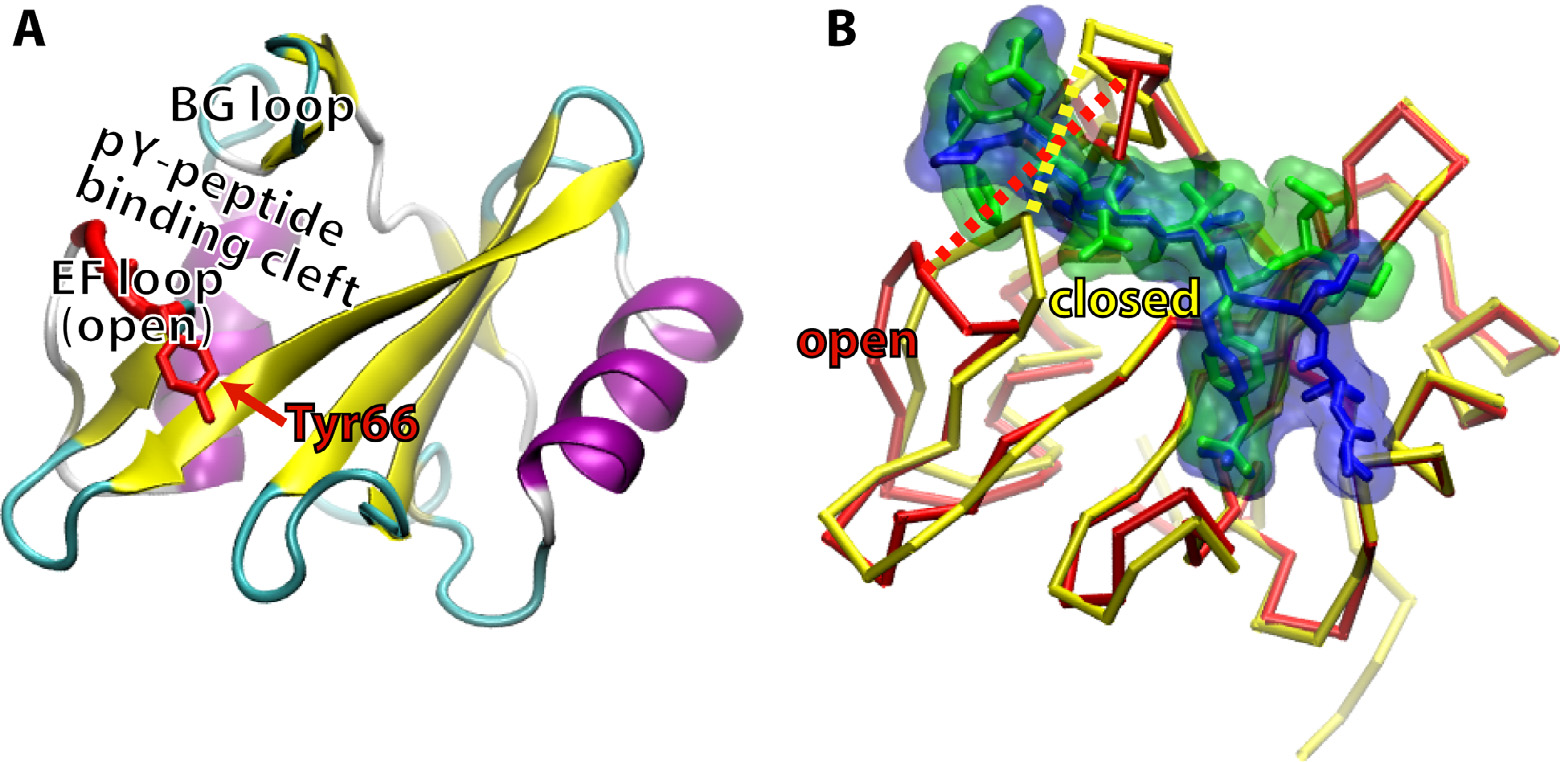
SHP-2 N-SH2 crystal structures. A) Crystal structure of isolated N-SH2 [PDB:1AYD] [9] showing the open pY-peptide binding cleft formed by the EF (red) and BG loops. B) Crystal structures of isolated N-SH2 [PDB:1AYD] (red), and N-SH2 in the full SHP-2 protein [PDB:2SHP] [4] (yellow). Dashed lines connect the C_α _atoms of Gly67 and Asn92. Also shown are pY-peptides from N-SH2 – pY-peptide complexes [blue from PDB:1AYA and green from PDB:1AYB] [9]. All structures were RMS aligned to the C_α _atoms of residues 6 to 55 of the SHP-2 crystal structure, which are structurally similar among the crystal structures. Molecular graphics were generated with VMD [45].

Crystal structures of N-SH2 alone, both with and without bound pY-peptides [9,10], show an open pY-peptide binding cleft between the EF loop (Tyr66-Gly67-Gly68) and the BG loop (Lys89-Glu90-Lys91-Asn92). This is in contrast to the crystal structure of the complete self-inhibited protein wherein the PTP-bound N-SH2's peptide-binding cleft is closed due to EF-loop motion and therefore unable to accommodate a pY-peptide (Figure 1). These structural studies, combined with biochemical evidence [5,6], imply that pY-peptide binding and disruption of the intramolecular N-SH2 – PTP interface, and hence activation of phosphatase activity, are normally coupled. Mutations at the protein – protein interface that disrupt the interface leading to the active form of the protein are associated with the congenital disease Noonan syndrome as well as childhood leukemias [11-13]. Accordingly, it may be anticipated that small-molecule inhibitors of either SHP-2 SH2 pY-peptide binding or PTP activity have the potential to serve as novel research tools and as potential precursors to therapeutics. To better understand the biochemical properties of the N-SH2 domain with the aim of developing N-SH2-specific inhibitors, we have used molecular dynamics (MD) simulations to investigate the closed-to-open transition of the N-SH2 pY-peptide binding cleft. Our data suggest that Tyr66 plays an important role in this conformational switching.

## Results and discussion

### N-SH2 pY-peptide binding cleft width in experimental crystal structures

The width of the pY-peptide binding cleft, formed by the EF (residues 66–68) and BG (residues 89–92) loops, can be characterized by the distance between the C_α _atoms of Gly67 and Asn92 (Figure 1b). In the crystal structure of the full SHP-2 protein [PDB:2SHP] [4], which consists of a 525-residue polypeptide comprising the two SH2 domains followed by the PTP domain and in the self-inhibited conformation, this distance is 8.2 Å and the cleft is in the closed conformation. In contrast, in the crystal structure of isolated N-SH2 without bound peptide [PDB:1AYD] [9] this distance is substantially longer at 14.3 Å, and reflects the fact that the cleft is open. Interestingly, in crystal structures of N-SH2 complexed with pY peptides, this distance assumes values of 14.8 Å ([PDB:1AYA], pY peptide = SVLpYTAVQP{NE}, amino acids in braces are missing coordinates in the crystal), 16.2 Å ([PDB:1AYB], {SP}GEpYVNIEF{GS}), and 14.4 Å ([PBD:1AYC], {D}GGpYMDMS{KGS}) [9]. The near-identical cleft widths in the isolated N-SH2 crystal structures both in the presence and absence of bound pY-peptide imply that, when not bound to PTP, N-SH2 adopts a conformation with an open pY-peptide binding cleft that is pre-arranged to bind pY-peptide. The open cleft conformation is thus more thermodynamically favorable than the closed conformation when N-SH2 is not bound to PTP, in contrast to the PTP-bound conformation in which the cleft is closed.

### Molecular dynamics simulations

Molecular dynamics (MD) simulations complement crystallographic structural data by providing a direct view of structural fluctuations and conformational changes at the nanosecond timescale. Consequently, transitions between two conformationally different crystal structures of the same protein can be directly probed via simulations. For example, if simulations starting from two different crystallographic conformations converge to one of the two conformations, it can be inferred that that sampled conformation is more stable and that there is a low or non-existent energy barrier for the transition. If, on the other hand, the crystallographic conformations are maintained, a barrier exists that prevents the transition on the time scale of the simulation.

To characterize the conformational properties of the N-SH2 pY-peptide binding cleft, all-atom nanosecond-scale explicit-solvent MD simulations at a temperature of 298 K and pressure of 1 atm were performed on isolated N-SH2 and the full SHP-2 protein, all in the absence of pY-peptide. The simulations included all protein and solvent degrees-of-freedom and thus can be used to probe the dynamic and thermodynamic properties of the solvated proteins at an atomic level-of-detail. Three systems were constructed using two x-ray crystal structures. Two systems were of the isolated N-SH2 domain, one having an open pY-peptide binding cleft as the starting conformation and the second having a closed pY-peptide binding cleft. The third system was of the full SHP-2 protein in which the cleft is closed. Table 1 summarizes the crystal structures used for system construction, the residue span, and the pY-peptide binding cleft conformation for the three systems, as well as the abbreviations used in the text to describe each system. All three systems were solvated with water molecules and neutralizing counterions, and were of a size such that a minimum of a 14-Å layer of water surrounded the protein on all sides. The resultant concentrations were 8 mM for the N-SH2 domain systems and 2 mM for the full SHP-2 system. The simulations were done using periodic boundary conditions to prevent boundary artifacts. Under periodic boundary conditions, the system sees its own image in all directions and when a molecule drifts out of one side of the system, it drifts back in from the opposite side. Further simulation details are described in the Methods section.

**Figure 2:**
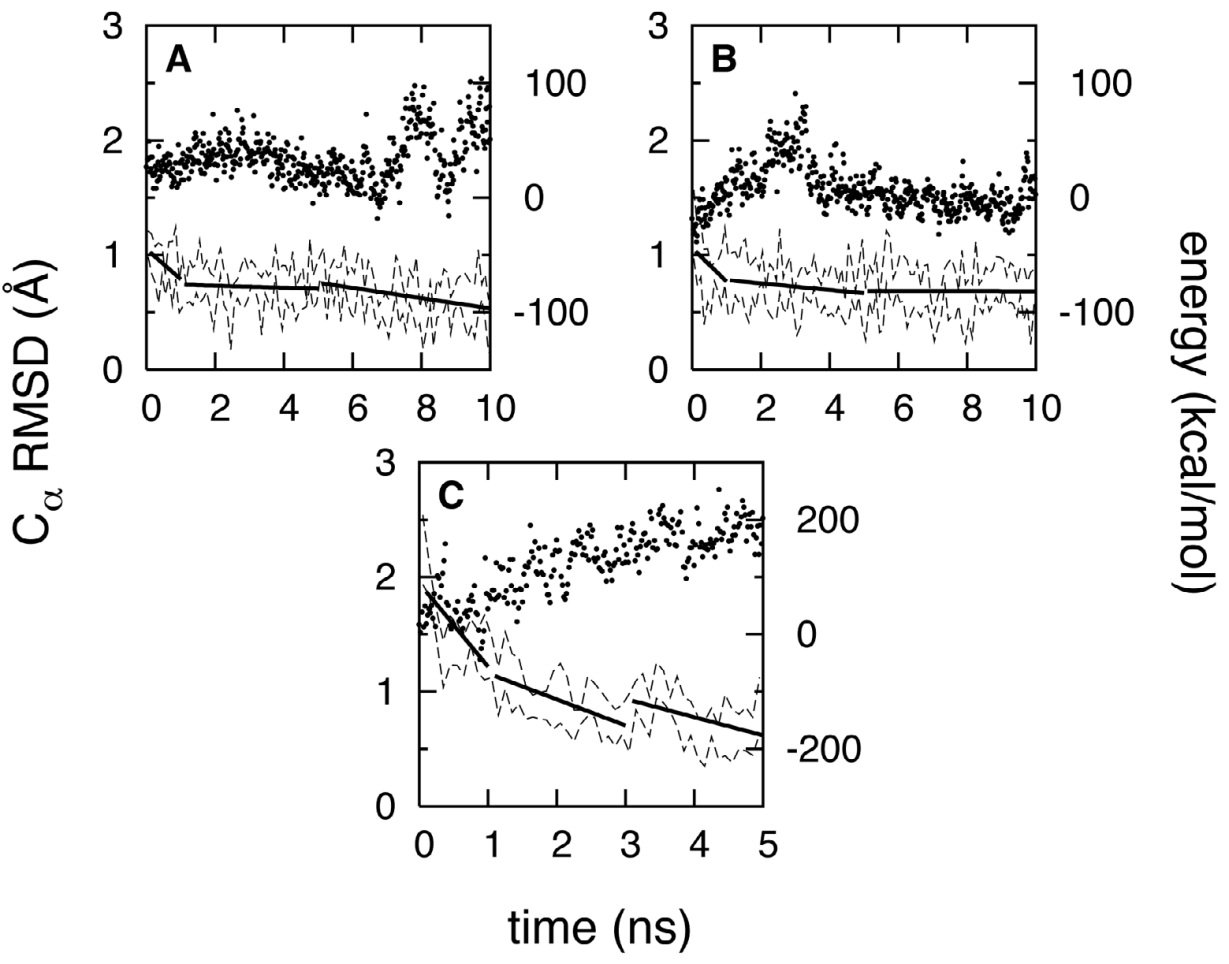
C_α _RMSD and average energy vs. time. Points represent the C_α _RMSD, solid lines are the least-squares fit to the average of the total system potential energy calculated in 0.1-ns blocks (<TOTE>_0.1 ns_), and dashed lines are located at <TOTE<_0.1 ns _± 2*SEM, where SEM is the standard-error-of-the-mean in the total energy for the 0.1-ns block as calculated from the average energies of the 50 successive 0.002-ns intervals in that block. The energies for each system have been offset by a constant for clarity. A) The N-SH2 system as constructed from the isolated N-SH2 crystal coordinates (1AYD:N-SH2). B) The N-SH2 system from the SHP-2 coordinates (2SHP:N-SH2). C) The entire SHP-2 protein using the SHP-2 coordinates (2SHP:SHP-2). RMSD values are calculated relative to the respective X-ray crystal coordinates after C_α_-atom least-squares alignment.

To test for equilibration of the MD simulations of the three systems, time courses for the C_α _root mean square deviations (RMSDs) relative to the respective crystal structures as well as average system energies were calculated. RMSD and average energy data correlate with how thoroughly the protein has relaxed in response to the surrounding environment and how thoroughly the environment has relaxed in response to the protein. The two 10-ns N-SH2 simulations reach plateaus in both average energy and RMSD by 3 ns (Figures 2a and 2b) and are thus well-equilibrated on the timescale of the simulations. The fluctuation in RMSD near the end of the 1AYD:N-SH2 simulation is solely due to motion in the N- and C-terminal residues, which are both on the opposite face of the protein as the binding cleft. The full SHP-2 system shows more limited convergence than the N-SH2 systems owing to its larger size, which both increases the timescale for equilibration and hinders longer sampling because of computational cost. Nonetheless, the simulation statistics are relatively constant during the 3–5 ns interval and show that the system has largely finished relaxation by 3 ns (Figure 2c). Given that the RMSD and average energy time courses reach equilibrium values by 3 ns for all three simulations, only molecular dynamics snapshots after the 3-ns time point were used for further analysis.

To investigate the conformational change in the pY-peptide binding cleft, average structures of N-SH2 from the 1AYD:N-SH2, 2SHP:N-SH2, and 2SHP:SHP-2 MD trajectories were calculated. Conformations sampled every 0.02-ns were RMS-aligned to their respective crystal structures using all atoms in residues 1–108. Average coordinates were then calculated for these aligned conformations for the 3–5 ns interval of the 2SHP:SHP-2 simulation and for the 3–5 ns and 5–10 ns intervals for the 1AYD:N-SH2 and 2SHP:N-SH2 simulations. The 1AYD:N-SH2 3–5 ns and 5–10 ns average structures both had large Gly67 C_α _– Asn92 C_α _distances, consistent with a wide pY-peptide binding cleft. Their values were 18.0 and 14.7 Å, as compared to the crystallographic distance of 14.3 Å. In contrast, distances from the two simulations initiated from the SHP-2 crystal structure were smaller, consistent with a narrow cleft, and similar to the crystallographic distance of 8.2 Å. These values were 9.9 Å for 2SHP:SHP-2:3–5 ns, 10.6 Å for 2SHP:N-SH2:3–5 ns, and 11.8 Å for 2SHP:N-SH2:5–10 ns. The upward drift in the 2SHP:N-SH2 values is accounted for by changes in the BG loop backbone conformation that bring it into consensus with the isolated N-SH2 crystal conformation, as detailed subsequently. Nonetheless, this N-SH2 domain system with the closed cleft, which was constructed using the full SHP-2 crystal structure, does not relax fully to the open cleft conformation despite the absence of the PTP domain.

**Figure 3:**
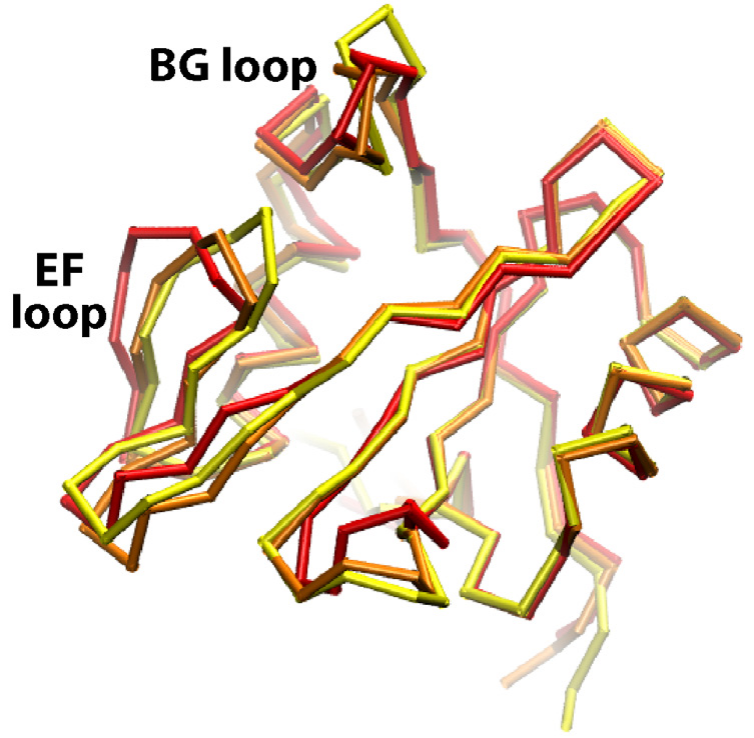
N-SH2 average structures from molecular dynamics simulations. Red: the 5–10 ns interval of the simulation of N-SH2 as started from the isolated N-SH2 crystal coordinates (1AYD:N-SH2:5–10 ns); Orange: the 5–10 ns interval of the simulation of N-SH2 as started from the full SHP-2 crystal coordinates (2SHP:N-SH2:5–10 ns); Yellow: the 3–5 ns interval of the simulation of the full SHP-2 protein (2SHP:SHP-2:3–5 ns).

Figure 3 illustrates the differences between average structures from all three simulations: 1AYD:N-SH2 has a different EF loop conformation than 2SHP:N-SH2 and 2SHP:SHP-2. This difference translates to a wider cleft for 1AYD:N-SH2, despite relaxation to very similar BG loop conformations for 1AYD:N-SH2 and 2SHP:N-SH2. The narrower cleft in the 2SHP:N-SH2 simulation demonstrates that the closed-to-open transition does not occur on the nanosecond timescale, though such transitions involving loop motion have been shown to occur on this timescale [14]. The inability of 2SHP:N-SH2 to fully relax to the open conformation over the course of 10 ns suggests the process is not diffusion controlled. Thus, while the crystallographic data imply that the open conformation is the preferred one (lower free-energy) for isolated N-SH2, the molecular dynamics predict an energetic barrier to the full closed-to-open transition.

**Figure 4:**
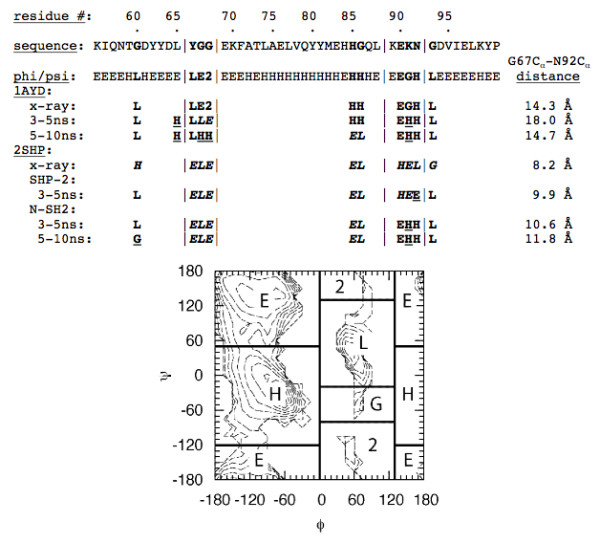
N-SH2 crystallographic and simulation-average secondary structures and Gly67 C_α _– Asn92 C_α _distances. Secondary-structure that is the same in all of the crystal and simulation structures is in plain text, secondary-structure corresponding to that in the isolated N-SH2 crystal is in bold, secondary-structure corresponding to that in the full SHP-2 crystal is in bold italics, and secondary structure corresponding to neither crystal structure is in bold underline. Vertical lines denote the spans of the EF (residues 66–68) and BG (residues 89–92) loops. The φ/ψ diagram was generated using the distribution of non-Gly, non-Pro backbone angles in a high-resolution x-ray crystal data set of 500 proteins [46]. The data were binned in 15° × 15° intervals, the natural logarithm of the histogram counts were taken, and contours have been drawn every 2 units. "E" = extended, "H" = helix, "L" = left-handed helix, "G" = gamma, and "2" = II'.

Backbone φ/ψ angles for residues 55 to 101 in the crystal structures and the average structures were calculated for a detailed comparison of the backbone conformations that correspond to the different pY-peptide binding-cleft widths. Residues were classified as being in extended (E), helical (H), left-handed helical (L), gamma (G), and II' (2) regions of φ/ψ space (Figure 4). Of the 47 residues in this span, 36 have the same secondary structure across both of the crystal structures and all five of the simulation average structures. The residues in which differences occur are 60, 65–68, 85–86, and 90–93, with 5 of these 11 being conformationally labile Gly residues. This set of 11 residues largely overlaps the residue ranges of the EF (residues 66–68) and BG (89–92) loops, which indicates the conformational variability of the two loops.

### BG loop conformation depends on N-SH2 – PTP binding

Analysis of the backbone conformation of the three systems' BG loops shows that the two simulations of N-SH2 converge to a common structure resembling that from the crystal of isolated N-SH2, whereas the crystallographic BG loop conformation in the full SHP-2 protein is maintained in the simulation of the full SHP-2 protein. The 1AYD:N-SH2 3–5 ns and 2SHP:N-SH2 3–5 ns average conformations assume the same secondary structure for residues 90–93, EHHL, one that is nearly the same as the isolated N-SH2 crystal structure and is maintained in the 5–10 ns interval average structures. In contrast, the 2SHP:SHP-2 3–5 ns average conformation maintains the 2SHP crystallographic secondary structure for Glu90 and Lys91. Thus, maintenance of the PTP-bound secondary structure of the N-SH2 BG loop (residues 89–92) requires that N-SH2 be bound to PTP, although residues in the vicinity of the loop (e.g. 85–86, Figure 4) can undergo conformational changes. In the absence of this protein – protein interface, the BG loop relaxes to a conformation consistent with the crystallographic conformation of the BG loop in isolated N-SH2. Interestingly, the N-SH2 BG loop has no direct contact with PTP in the SHP-2 crystal structure. The difference in BG loop conformations between the PTP-bound and unbound N-SH2 states implies conformational coupling: a preference for a particular BG loop conformation is induced by interaction of a different portion of the N-SH2 domain with the PTP-domain, thereby communicating the presence of the PTP-domain to the BG loop.

### EF loop conformation correlates with the Tyr66 backbone conformation

Contrasting behavior is observed in the EF loop (residues 66–68, sequence YGG) where, unlike the BG loop in the N-SH2 simulations, structural convergence does not occur. In all three simulations the backbone conformation for Tyr66 is retained relative to that of the respective crystals, while those of the two glycine residues (67 and 68) show more conformational flexibility (Figure 4). For Tyr66, the 1AYD:N-SH2 average structures maintain the crystallographic left-handed helical backbone conformation, L, whereas the 2SHP:N-SH2 and 2SHP:SHP-2 average structures maintain the crystallographic extended backbone conformation, E. The adjacent Leu65 does exhibit an E-to-H transition in one of the two N-SH2 domain simulations, but this is a small conformational change as the crystallographic φ/ψ values are -113/61 for this residue such that only a 2° change in the ψ angle is required to effect the change in classification. These results indicate Tyr66 to be the key difference in the two N-SH2-domain simulations, since it is the only non-Gly residue whose backbone conformation is both different in the two crystals and does not show conformational convergence in the simulations.

Supporting the important role of Tyr66 in dictating the conformation of the EF loop is the correlation between the cleft-width, as measured by the Gly67 C_α _– Asn92 C_α _distance (Figure 4), and the Tyr66 backbone conformation. When Tyr66 is in the extended region of φ/ψ space, the cleft is closed and when Tyr66 is in the left-handed helix region of φ/ψ space, the cleft is open. Thus although six residues among the seven that compose the EF and BG loops have different conformations in the two crystal structures, the backbone conformation of Tyr66 appears to be a key factor in the closed-to-open conformational change in the pY-peptide binding cleft. This observation motivated additional calculations to validate Tyr66 as a conformational switch dictating the structure of the EF loop.

### Deep free-energy minima keep the Tyr66 backbone locked in position

To obtain a quantitative estimate of the energetics associated with the conformational change in Tyr66, free-energy simulations were carried out using the final N-SH2 domain conformations from the 1AYD:N-SH2 and 2SHP:N-SH2 simulations. By applying restraining potentials to the φ/ψ dihedrals of Tyr66, Tyr66 conformations in the neighborhood of these final conformations were preferentially sampled while averaging over all other protein degrees of freedom. The data were appropriately re-weighted and combined to yield effective free-energy surfaces, or "potentials-of-mean-force" (PMFs), as a function of the Tyr66 backbone conformation. These PMFs reveal the free-energy barriers to changes in Tyr66 backbone conformation for solvated N-SH2 at room temperature and atmospheric pressure.

**Figure 5:**
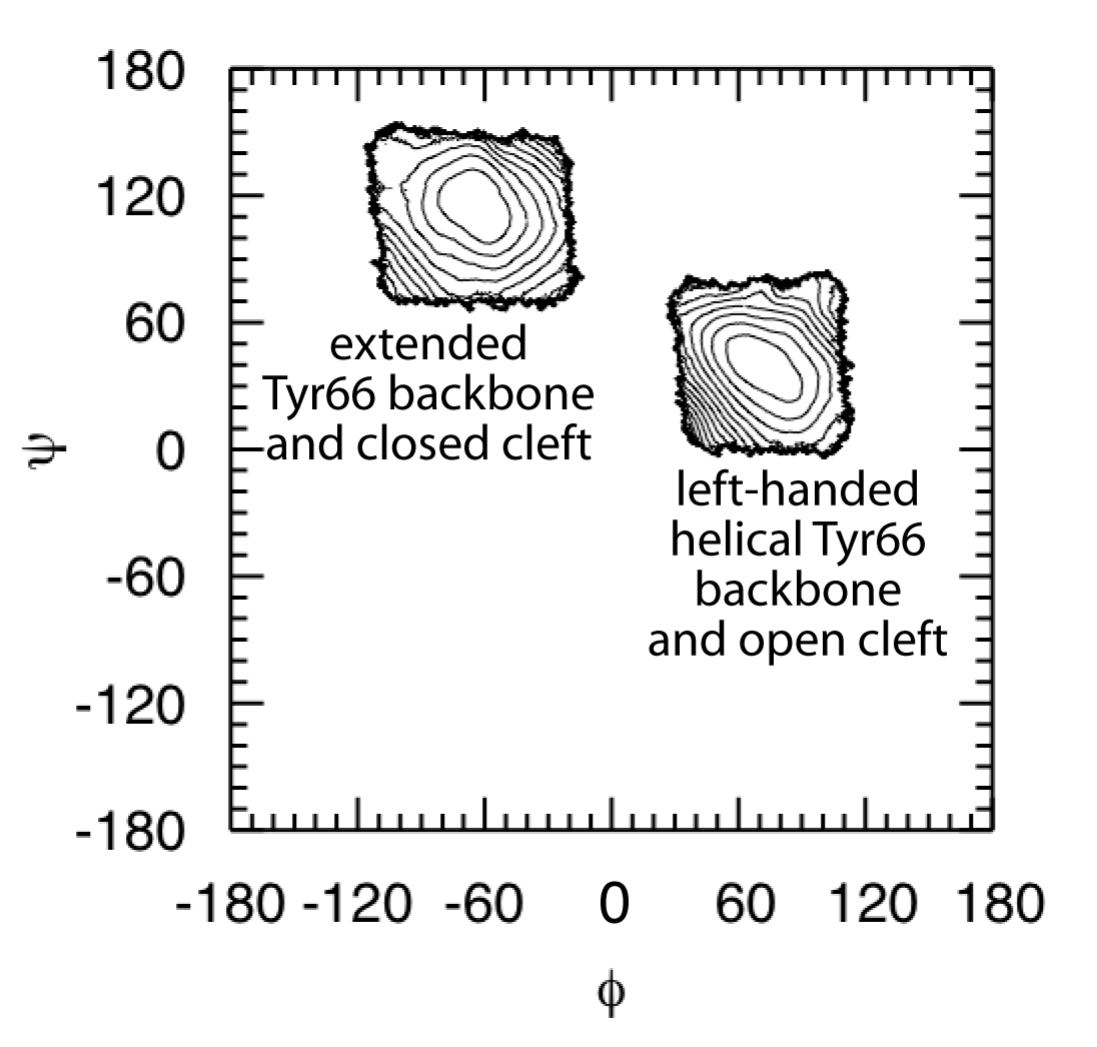
N-SH2 Tyr66 φ/ψ free-energy surfaces at 298 K and 1 atm. The open cleft conformation correlates with left-handed helical Tyr66 φ/ψ geometry and the closed cleft conformation correlates with extended Tyr66 φ/ψ geometry. Contours are every 1 kcal/mol.

From the PMF data, displacements of only 20 to 40 degrees in either the φ or ψ coordinate of the Tyr66 backbone translate to increases in free energy of 3 or more kcal/mol (Figure 5). Thus, in the open cleft state, Tyr66 is constrained to a small portion of φ/ψ space corresponding to a left-handed helical conformation, and in the closed cleft state Tyr66 is constrained to a small portion of φ/ψ space corresponding to an extended conformation. These deep and well-defined free-energy minima serve to lock Tyr66 into a particular backbone conformation, thereby explaining the lack of EF loop relaxation to a consensus conformation in the two 10-ns molecular dynamics trajectories of isolated N-SH2 (1AYD:N-SH2 and 2SHP:N-SH2).

**Figure 6:**
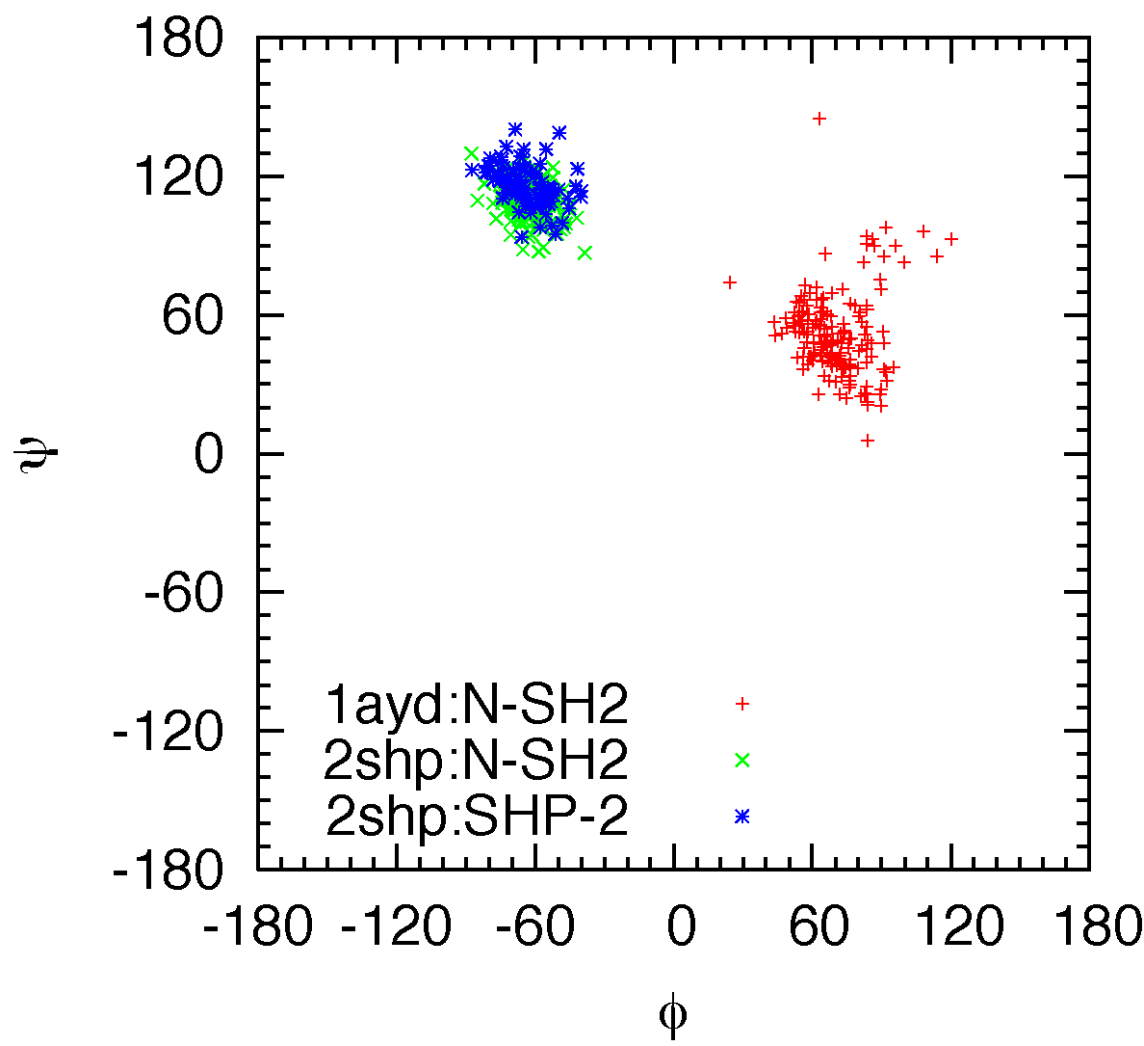
Tyr66 φ/ψ distributions in the three simulations. Data are taken at 0.02-ns intervals and span the full lengths of the simulations, which were 10 ns for 1AYD:N-SH2 and 2SHP:N-SH2, and 5 ns for 2SHP:SHP-2.

If indeed Tyr66 is the key residue that modulates the closed-to-open transition of the pY-peptide binding cleft, the fact that it is constrained to two small regions of φ/ψ space allows it to act like a molecular switch. Intermediate states become very unlikely because they are high in free energy and therefore the EF loop is in either the closed or open conformation depending on the Tyr66 backbone conformation, and not in some intermediate state. The PMF data are corroborated by the nanosecond-scale MD simulations. In the case of 2SHP:N-SH2 and 2SHP:SHP-2, Tyr66 only samples φ/ψ conformations confined to the "closed" contours illustrated in Figure 5 while 1AYD:N-SH2 samples conformations confined to the "open" contours (Figure 6).

### A change in the Tyr66 backbone conformation induces a conformational change in the EF loop

**Figure 7:**
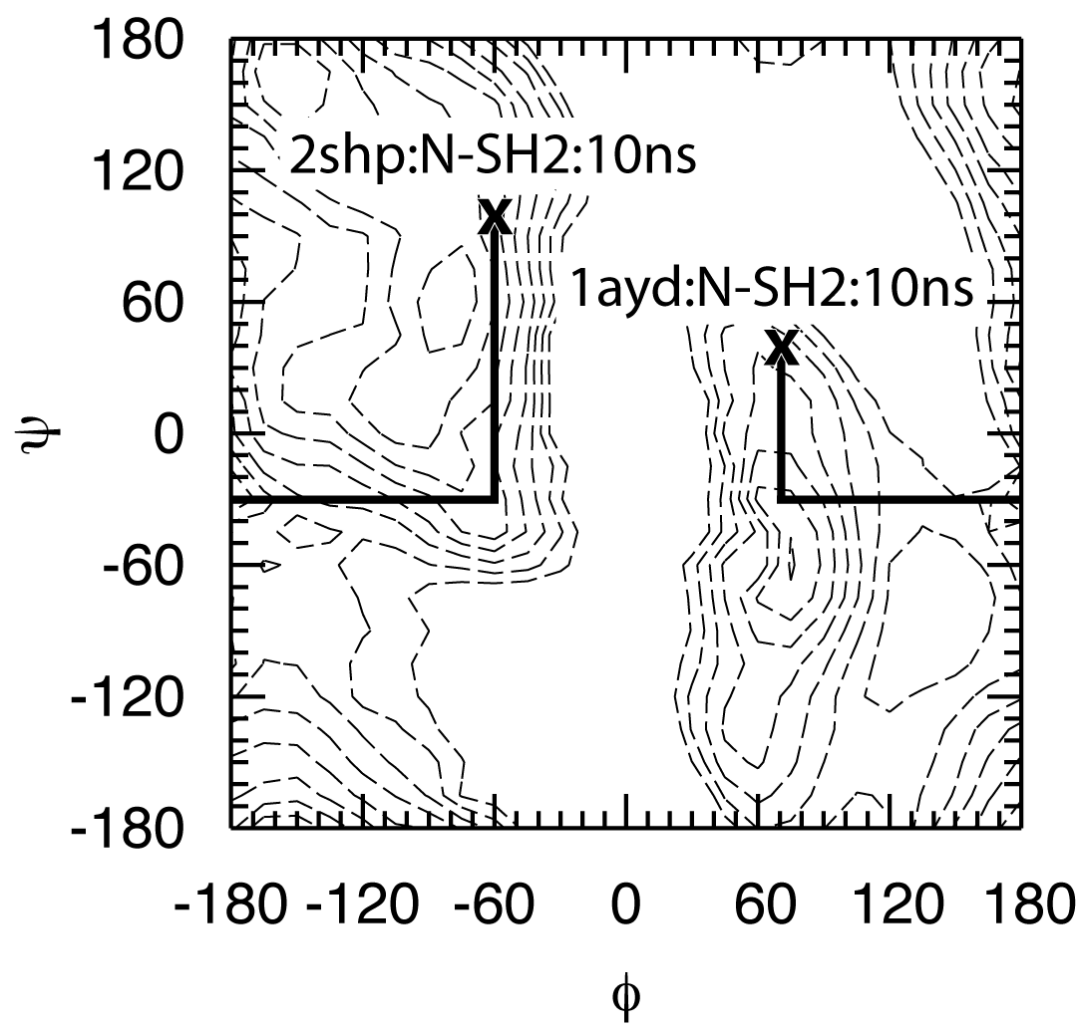
Reaction path for the interconversion of Tyr66 between the extended (E) and left-handed helical regions (L) of φ/ψ space. The adiabatic *in vacuo *energy surface for the alanine dipeptide as modeled by the CHARMM all-atom force field is represented as contours, with isoenergetic lines every 1 kcal/mol for the first (lowest) 8 kcal/mol.

To test the ability of Tyr66 to act as a conformational switch in the closed-to-open transition of the SHP-2 N-SH2 pY-peptide binding cleft, simulations were performed in which conversion between the left-handed helical (L) and extended (E) Tyr66 backbone conformations was induced, both in the forward and backward directions. Starting with the final conformation from the 10-ns 2SHP:N-SH2 simulation, in which the Tyr66 backbone was in the "E" conformation, an E-to-L transition was induced over the course of 1 ns using restraining potentials on the Tyr66 φ and ψ angles and following the reaction path shown in Figure 7. Similarly, starting with the final conformation from the 10-ns 1AYD:N-SH2 simulation, in which the Tyr66 backbone was in the "L" conformation, an L-to-E transition was induced. The basis for the selection of this path, versus the more direct path between the two states, was its energetic accessibility as judged by the conformational energies of the CHARMM backbone force field as included in Figure 7. These two sets of simulations directly test whether an induced change in the backbone of Tyr66 can cause a transition between the closed and open states of the pY-peptide binding cleft.

**Figure 8:**
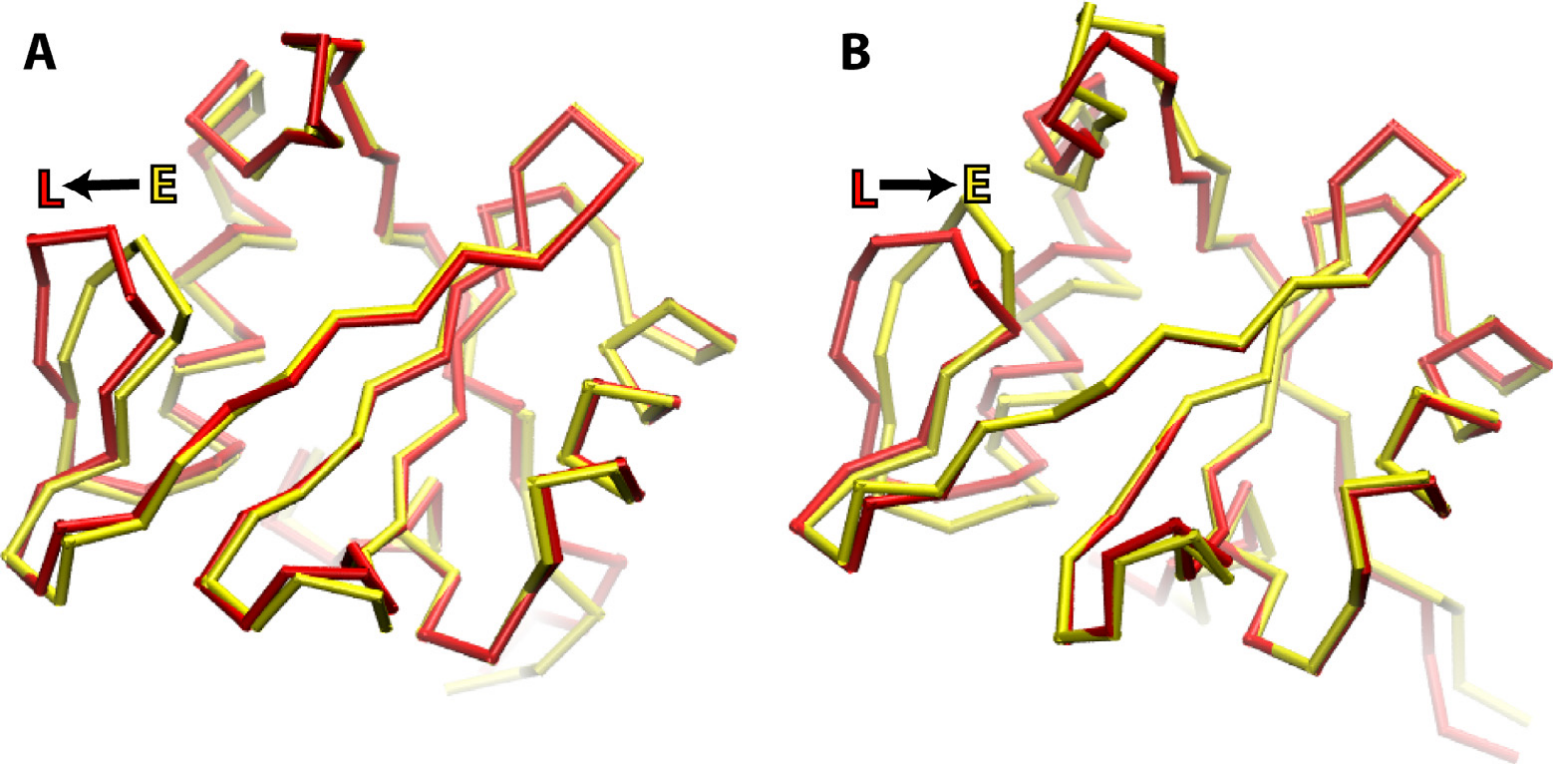
Average structures from the first and last 10-ps intervals of the 1-ns Tyr66 backbone E-to-L and L-to-E induced transitions. Structures with the Tyr66 backbone in the "E" conformation are in yellow and in the "L" conformation are in red. Average structures were calculated after all-atom RMS alignment of all snapshots with respect to the relative crystal structure. The average structures have been RMS aligned to each other using the C_α _atoms of the structurally invariant residues 6 to 55. A) The E-to-L transition as performed from the last snapshot from the 10-ns 2SHP:N-SH2 molecular dynamics simulations. B) The L-to-E transition as performed from the last snapshot from the 10-ns 1AYD:N-SH2 molecular dynamics simulation.

Average conformations calculated from 50 snapshots spaced 0.2 ps apart, both at the beginning and end of each of the two induced transitions, reveal the direct effect that the E-to-L and L-to-E Tyr66 conformational changes have on pY-peptide binding cleft width. The Tyr66 E-to-L transition causes a closed-to-open transition in the binding cleft and the L-to-E transition causes an open-to-closed binding cleft transition. After alignment of the pair of average structures from the E-to-L transition using the C_α _atoms of the structurally invariant residues 6 to 55 (C_α _RMSD 0.44 Å), the C_α _atoms of all three residues in the EF loop have moved away from the center of the cleft (Figure 8a). The average position of Tyr66 C_α _has been displaced by 2.7 Å, Gly67 by 2.8 Å, and Gly68 by 4.7 Å. The same alignment using the average structures from the endpoints of the L-to-E transition (0.37 Å C_α _RMSD for residues 6 to 55) reveals the opposite motion, with the C_α _atoms of all three residues moving toward the center of the cleft (Figure 8b). The average position of Tyr66 C_α _has been displaced by 2.4 Å, Gly67 by 1.8 Å, and Gly68 by 5.5 Å. These data provide direct evidence that Tyr66, and in particular its backbone conformation, acts as a conformational switch that can control the relative positioning of the EF loop and thereby effect the closed-to-open binding cleft transition.

### The role of the Tyr66 sidechain

**Figure 9:**
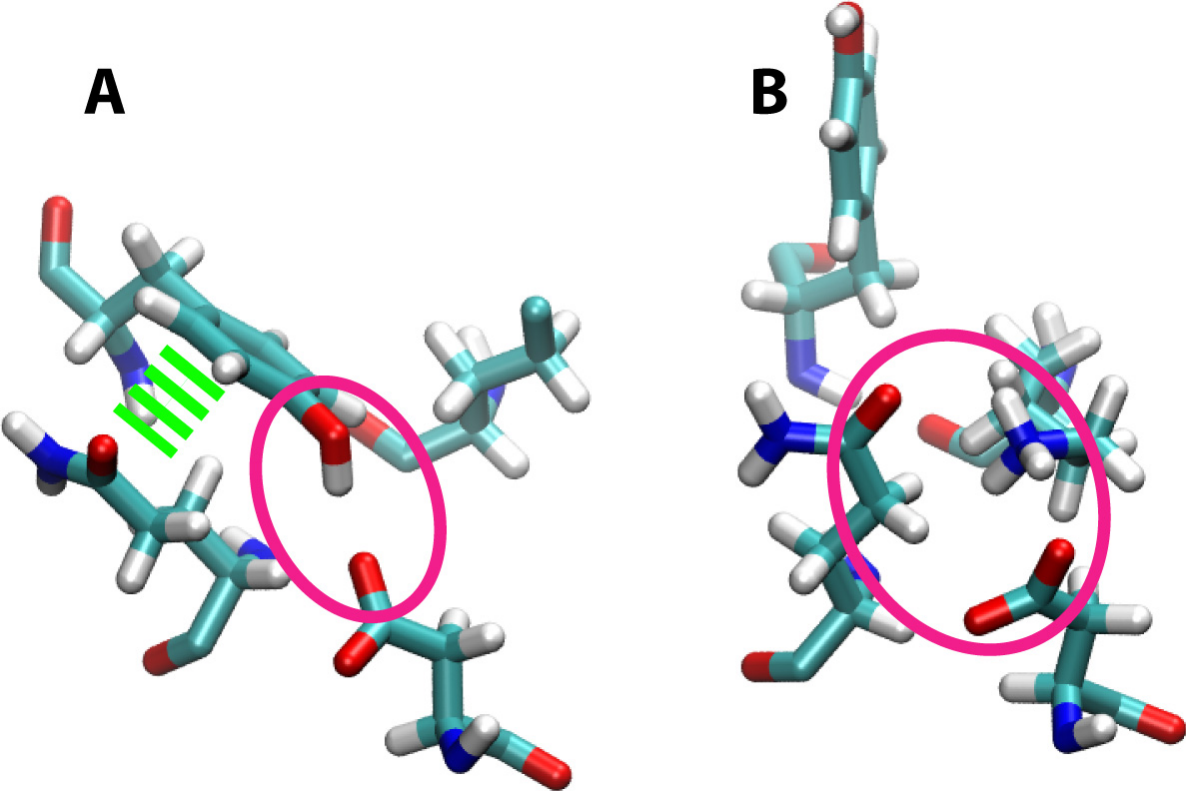
Crystallographic conformations of Asp40, Lys55, Gln57, and Tyr66. A) Conformation in the isolated N-SH2 domain [PDB:1AYD]. B) Conformation in the full SHP-2 protein [PDB:2SHP]. π-stacking is represented by green dashes and hydrogen-bond/salt-bridge interactions are circled in pink. The C_ε _and N atoms of the Lys55 sidechain are missing in 1AYD.

Visual analysis indicates sidechain – sidechain interactions involving Tyr66 help to anchor the open form of the EF loop and pull the sidechain away from the entrance to the wide pY-peptide binding cleft. In contrast, in the closed form this sidechain has no interactions with other parts of the protein and partially occludes the entrance to the narrow cleft (Figure 9). This difference in sidechain positioning and interactions is due not only to the differences in Tyr66 backbone conformation, and hence EF loop position, but also to a difference in the χ_1 _dihedral of the residue's sidechain. Due to the sp^2^-hybridized C_γ _and the internal symmetry of the sidechain, Tyr66 has two equivalent minima at χ_2 _= ± 90°. Therefore, when the sidechain is in a local minimum, only φ, ψ and χ_1 _are required to denote its geometry.

For a three-fold rotation like χ_1_, local minima exist at idealized values of -60°, 60°, and 180°. Crystallographically, in the open conformation [PDB:1AYD] χ_1_/χ_2 _= -65.2°/88.7°, and in the closed conformation [PDB:2SHP] χ_1_/χ_2 _= -165.3°/-83.4°. Thus, with the cleft open (Figure 9a) χ_1 _exists in the -60° minimum and with the cleft closed (Figure 9b) χ_1 _is in the 180° minimum. The combination of the open EF loop conformation due to the left-handed helical Tyr66 backbone and the χ_1 _angle in the -60° minimum pins the Tyr66 sidechain to the surface of the N-SH2 domain (Figure 1a). Its close proximity to other surface amino acids enables the formation of hydrogen bonding and π-stacking with two other sidechains, thereby reinforcing the open conformation. In particular, in the crystallographic open form Tyr66 acts as a hydrogen-bond donor with respect to Asp40 and participates in π-stacking with Gln57 (Figure 9a).

The interactions involving Tyr66 appear to be unique to SHP-2 and the closely related SHP-1. BLAST [15] alignment of the SHP-2 N-SH2 sequence shows that other homologous human SH2 domains do not have a tyrosine residue at this position, though they sometimes possess one or both subsequent glycine residues, and BLAST search of the 11-residue span with the YGG sequence in its middle yields only SHP-2 and SHP-1. Such lack of homology suggests a potentially unique role of Tyr66 in the SHPs.

**Figure 10:**
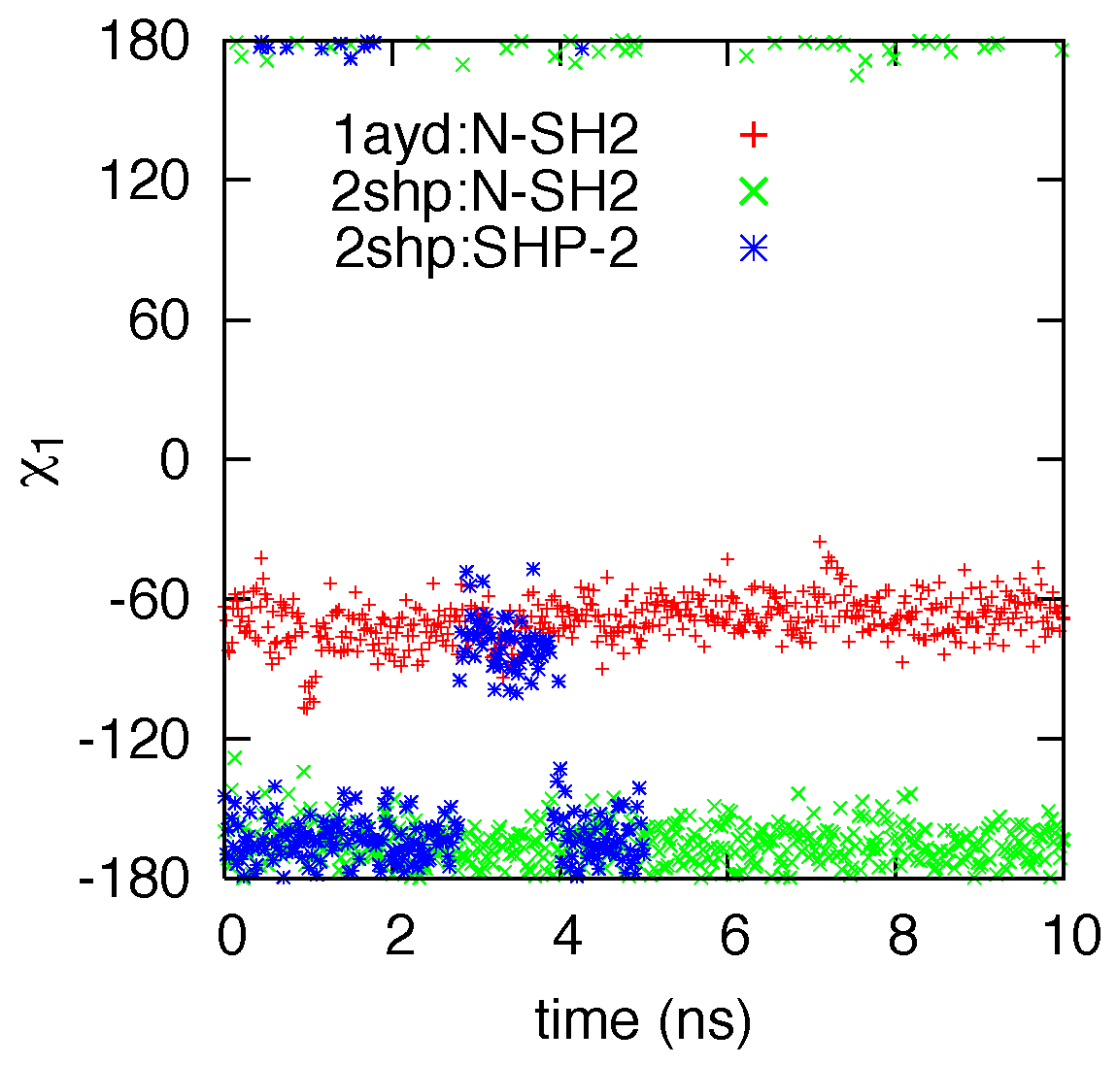
Tyr66 χ_1 _dihedral angle values as a function of time.

The stability of the χ_1 _angle and its correlation with the state of the EF loop is evidenced in the MD trajectories. The trajectory of isolated N-SH2 in which the EF loop is in the open conformation (1AYD:N-SH2) maintains crystallographically-consistent χ_1 _values in the -60° minimum. Likewise, the trajectories of the N-SH2 domain in which the EF loop is in the closed conformation (2SHP:N-SH2 and 2SHP:SHP-2) sample χ_1 _values in the 180° minimum (Figure 10). Surprisingly, unlike the trajectories of the isolated N-SH2 domain in which χ_1 _exclusively samples the crystallographic minimum, the trajectory of the full SHP-2 protein sees a fluctuation from its crystallographic minimum to the other crystallographic minimum and back again. This conformational fluctuation suggests that though χ_1 _is involved in the closed-to-open transition, it is comparatively labile with respect to the backbone conformation of Tyr66 φ/ψ, which by itself is able to cause EF loop motion between the closed and open states and stays locked in either the extended or left-handed helical state for the full duration of the 10-ns N-SH2 and 5-ns SHP-2 molecular dynamics trajectories. This sidechain mobility is also evident in the simulation in which the open-to-closed transition is induced. There, as forces applied to the Tyr66 backbone induce the L-to-E backbone transition, χ_1 _spontaneously transitions from the -60° minimum to the 180° minimum.

The simulation of isolated N-SH2 started from the open-cleft isolated N-SH2 crystal structure shows the Tyr66 – Asn40 sidechain – sidechain hydrogen bonding and the Tyr66 – Gln57 π-stacking as in that crystal structure. Additionally, Tyr66 – Lys55 sidechain – sidechain hydrogen bonding is observed; this interaction is consistent with the crystal structure because crystallographically these sidechains are in close proximity, though atoms beyond Lys C_δ _are missing crystal coordinates, presumably due to conformational flexibility (Figure 9a). The simulation data also reveal the fluctuating nature of these sidechain – sidechain interactions. For example, Gln57 alternates between π-stacking and hydrogen bonding with the Tyr66 sidechain. Asp40 during the first 5-ns goes back and forth between direct and water-mediated hydrogen bonding with Tyr66 and drifts out of hydrogen bonding distance during the last 5-ns. And Lys55 forms both direct and water-mediated hydrogen bonding networks with the Tyr66 sidechain over the full 10 ns. Taken separately, the Tyr66 interactions with the three sidechains from Asp40, Lys55, and Gln57 are transient and therefore weak. However, when combined these residues anchor the Tyr66 sidechain to the surface of the domain and away from the opening to the pY-peptide binding cleft.

When the Tyr66 sidechain is freed from interaction with these three sidechains and can partially occlude the cleft, the three sidechains themselves form a hydrogen-bonding complex. In this complex the sidechain of Lys55 serves as a hydrogen-bond donor to the Asp40 and Gln57 sidechains both in the SHP-2 crystal structure and the simulations (Figure 9b). As with the Tyr66 sidechain interactions in the open-cleft form, direct interactions between these three sidechains are transient in the MD simulations started from the open-cleft N-SH2 structure. In the case of 2SHP:N-SH2, the Asp40 – Lys55 hydrogen bond fluctuates during the first 5 ns whereas it remains stably formed during the last 5 ns. The Gln57 – Lys55 hydrogen bond present at the beginning of this trajectory is lost when a water molecule intercedes, but the interaction among the three sidechains is maintained by the formation of a hydrogen bond between the Gln57 and Asp40 sidechains. Likewise, there is an interaction triad involving Asp40, Lys55, and Gln57 in the case of 2SHP:SHP-2. However, during the 3–4 ns interval χ_1 _fluctuates to the -60° minimum (Figure 10), allowing the Tyr66 sidechain to form a persistent hydrophobic interaction with the aliphatic sidechain atoms of Lys55 and transient hydrophobic and hydrogen bonding interactions with the sidechain of Gln57. After this interval, χ_1 _reverts to its crystallographic conformation in the 180° minimum. Thus, when the EF loop is in its closed conformation, the fully solvent-exposed Tyr66 sidechain is sufficiently mobile as to be able to sample the open-cleft χ_1 _conformation that puts it in contact with other amino acids on the N-SH2 surface and away from the entrance to the binding cleft.

In summary, weak solvent-exposed interactions involving the sidechains of Tyr66, Asp40, Lys55, and Gln57 are important to the sidechain conformation of Tyr66. When the cleft is open, Asp40, Lys55, and Gln57 all interact with the Tyr66 sidechain and pin it to the surface of the protein and away from the cleft opening. Conversely, when the cleft is closed, Asp40, Lys55, and Gln57 form an interaction triad, freeing the Tyr66 sidechain from being tied to the surface of the protein and allowing it to become fully solvent exposed and to partially occlude the cleft.

## Conclusion

The SHP-2 N-SH2 Tyr66 backbone exists in two well-defined conformations, extended or left-handed helical, owing to the presence of free-energy barriers that encircle small low-free-energy regions in φ/ψ space. Conversion from one backbone conformation to the other leads to motion of the N-SH2 EF loop and a change in the width of the pY-peptide binding cleft between the EF and BG loops. Thus, with two well-defined positions that control the position of the EF loop, Tyr66 acts as a conformational switch that determines the state of the binding cleft as either closed or open. With an extended backbone conformation at Tyr66, the binding cleft is closed and consistent with the self-inhibited SHP-2 conformation in which N-SH2 and PTP form a protein – protein interface. Conversely, with a left-handed helical backbone conformation at Tyr66, the binding cleft becomes open, N-SH2 is able to localize SHP-2 to a particular region of the intracellular space via pY-peptide binding, and the PTP catalytic site is exposed and phosphatase activity increases.

Such conformational switching is in contrast to another possible scenario in which a continuum of Tyr66 backbone, and hence EF loop, conformations exists. In such a case, rigidification of the binding cleft into either the closed or open state would be associated with an entropic cost and hence a free-energy penalty. pY-peptide binding necessarily must be relatively weak so as to be readily reversible, as required for its role in intracellular signal transduction [16]. This is reflected in the interface between N-SH2 and pY-peptides, wherein only the portion of the pY-peptide that is in between the BG and EF loops is buried to a significant extent. The rest of the pY-peptide forms a relatively flat interface with N-SH2, in contrast to strong protein – peptide binding interactions in which a significant portion of peptide is deeply buried in the protein [17]. If in addition to burying only a small portion of the peptide, significant loop entropy must also be overcome for pY-peptide binding, the interaction would be weaker still and this might negatively impact the ability of N-SH2 to mediate SHP-2 localization and activation. Consequently, we predict a Tyr66Gly mutation would significantly weaken the ability of N-SH2 to bind pY-peptides.

A possible binding mechanism is suggested by the transition in Tyr66 χ_1 _conformation in the simulation of the full self-inhibited SHP-2 protein. The sidechain transition from the crystallographic closed-cleft χ_1 _minimum to the open-cleft χ_1 _minimum and back again shows that this sidechain does not always occlude the entrance to the binding cleft when N-SH2 is bound to PTP. pY-peptide could form an initial contact between its phosphotyrosine and immediately adjacent residues and the surface of N-SH2 corresponding to the pY, pY-1, and pY+1 positions. This initial contact would then be followed by secondary pY-peptide contact with the EF and BG loops that was facilitated by spontaneous fluctuation of the Tyr66 sidechain away from its cleft-occluding position and into contact with Lys55 and Gln57. The secondary contact would serve to open the pY-peptide binding cleft, thereby securing the full N-SH2 – pY-peptide binding interaction, inducing conformational changes in the EF and BG loops (neither of which have direct contacts with the PTP domain), and leading to loss of the N-SH2 – PTP interface and exposure of the PTP catalytic site. At this point, the Tyr66 sidechain would be pinned away from the binding cleft entrance by the formation of interactions with Asp40 as well as Lys55 and Gln57.

Such interactions involving Tyr66 as a conformational switch also suggest a possible role for this and surrounding residues in the regulation of SHP activity and cellular localization. Interactions with environmental factors, such as ions or other peptides, that impact the conformation of Tyr66 would facilitate either opening or closing of the pY-peptide binding cleft. Such changes would thereby favor or disfavor binding leading to alterations in the phosphatase activity and to cellular localization. While such a role is speculative, the present results suggest mutations that may be used to experimentally investigate this hypothesis. Mutations at Tyr66 to amino acids with large sidechains that cannot form hydrogen-bond/salt-bridge and/or π interactions may destabilize the open conformation in which Tyr66 sidechain interactions help to keep the sidechain away from the binding cleft entrance, thereby weakening peptide binding. Similarly, mutation of Asp40, Lys55, and/or Gln57 to Ala would weaken the ability of these residues to pin Tyr66 to the protein surface and away from the binding site entrance, and again adversely affect pY-peptide binding, and therefore cellular localization and phosphatase activation.

The fact that the EF loop position is well-defined is fortuitous from an inhibitor-design perspective, as the open conformation, which is thermodynamically more favorable based on the crystallographic data, can be targeted using structure-based inhibitor-design methods without complications due to the effects of EF-loop entropy and induced fit on small-molecule binding. Recent work on the pY-peptide binding-specificities of SHP-1 and SHP-2 suggests that the EF loop – BG loop cleft confers specificity, thus it may be possible to develop inhibitors targeting the cleft that not only block pY-peptide binding, but also preferentially do so for SHP-2 versus SHP-1 [18]. Such inhibitors can serve as research tools in the investigation of leukemia- and Noonan syndrome-associated SHP-2 mutants that have lost the ability to form the N-SH2 – PTP interface characteristic of the self-inhibited wild-type SHP-2. These mutants have constitutive high phosphatase activity [19,20] and increased N-SH2 pY-peptide binding affinity [21,22]. SHP-2 N-SH2 inhibitors would be useful in probing the importance of increased localization versus increased phosphatase activity in these mutants, and would also have the potential to serve as precursors to therapeutics.

## Methods

### Construction of missing crystal coordinates

The SHP-2 crystal structure was prepared for MD simulations by constructing missing residues, reverting mutated residues to their wild-type identities, and optimally determining hydrogen atom positions and Asn, Gln, and His sidechain orientations. The crystal structure of the entire self-inhibited SHP-2 phosphatase [PDB:2SHP], lacks coordinates for residues 1, 156 to 160, 236 to 245, 295 to 301, and 313 to 323. The crystal structure of the catalytic domain of SHP-1 [23], the closest homolog of SHP-2, could not be used for modeling the missing SHP-2 residues due to low homology and gap regions after PTP sequence alignment and the lack of Protein Data Bank coordinates for the SHP-1 SH2 domains. Instead, loop modeling [24] as implemented in the MODELLER 8v1 software [25] and consisting of 100 independent simulated-annealing runs for each span of missing residues was applied. This was done in an iterative fashion, such that residue 1 was modeled first, and the best conformation based on MODELLER energy was chosen as the starting model for modeling of the 156 to 160 residue span, and so on. Missing residue spans were sufficiently distant from each other in the structure so as to allow this approach.

After completion of loop modeling, the missing sidechain of Lys235 was reconstructed in its most probable conformation based on protein structural database statistics [26]. Also, the three mutations in the crystal structure – Thr2Lys, Phe41Leu, and Phe513Ser – were reverted to their wild-type identities. Like Lys235, Thr2 and Phe41 sidechains were built using database statistics [26]. The Phe513 sidechain was built using χ angles from the SHP-1 catalytic domain crystal structure's Phe509, which is in the middle of a α-helix whose sequence is conserved between SHP-1 and SHP-2. In the SHP-2 crystal structure, Phe513Ser results in a cavity that is occupied by a detergent molecule; reversion to the wild-type residue by modeling fills this cavity. Using version c32b1 of the CHARMM software [27], hydrogen atoms were constructed using CHARMM force field geometries [28], positional constraints were placed on all heavy atoms except those of the four sidechains, all force-field energy terms excluding electrostatics were turned on, and using force-field default non-bonded cutoffs the geometry was optimized with 1000 steps of steepest descent [29] followed by 1000 steps of conjugate gradient minimization [30]. Finally, the Reduce software [31] was applied to determine the optimal placement of hydrogen atoms, which includes optimization of the adjustable groups OH, SH, NH3+, Met-CH3, and Asn, Gln and His sidechains by rotation or flipping and, for the His sidechain, determination of protonation at the δ *vs*. ε position.

The crystal structure of the N-SH2 domain [PDB:1AYD] was similarly treated to prepare it for solvation and molecular dynamics. First, the Reduce software was applied. Second, Met3 was deleted, as this crystal structure contains only residues 3 to 103, with a Ser3Met mutation. Missing heavy atom positions were placed using coordinates from the complete SHP-2 modified crystal structure's N-SH2 domain after RMS alignment using the C_α _atoms of residues 4 to 103, and missing hydrogen positions were built using force field geometries. Heavy atoms with SHP-2 coordinates were harmonically restrained with a force constant of 2 kcal*mol^-1^*Å^-2 ^and other heavy atoms positions were constrained not to change. The system was then minimized with 5000 steps of steepest descent and 5000 steps of conjugate gradient minimization and then visualized to ensure that all geometries involving atoms whose coordinates were taken from the modified complete SHP-2 crystal structure were consistent with those seen in crystal surveys [26].

Using the two modified crystal structures, three polypeptides were created for subsequent molecular dynamics simulations. The first was the complete SHP-2 protein. The second was the N-SH2 domain from the complete SHP-2 protein and generated by deletion of all but residues 1–108. The last was the N-SH2 domain as created from the N-SH2 domain-only crystal structure. All polypeptides had positively charged amino termini and negatively charged carboxy termini.

### Construction of the solvated systems

The CHARMM all-atom force field [28] in conjunction with the grid-based "CMAP" term for accurate backbone energetics [32] was used to model the polypeptide, water molecules were represented with the TIP3P water model [33] as modified for the CHARMM all-atom force field [34], and the SHAKE algorithm [35] was used to constrain all bonds to hydrogen atoms to their equilibrium lengths. Crystallographic water molecules located within 5 Å of the polypeptide were maintained and the composite polypeptide/crystallographic water system was minimized with 5000 steps of steepest-descent minimization [29] followed by 5000 steps of conjugate-gradient minimization [30]. Next, a pre-equilibrated truncated octahedron of water molecules was super-imposed on the polypeptide such that the polypeptide was at its center. The size of the truncated octahedron was chosen to ensure at least a 14 Å layer of water between protein atoms and the nearest edge. All added water molecules within 4.1 Å of either the polypeptide or crystallographic water molecules were deleted. Neutralizing counterions were placed by random selection of added water molecules and their replacement by sodium ions, with subsequent visualization to confirm that no ions were placed within the protein. Three sodium ions were thus placed for the full SHP-2 protein, and one sodium ion for each of the N-SH2 systems. Periodic boundary conditions were applied [36], and Coulomb interactions were treated with the particle mesh Ewald method [37], with a real-space cutoff of 10 Å, a κ value of 0.32, order six B-spline interpolation, and a grid spacing of ~1 Å while Lennard-Jones interactions were truncated with force-switching [38] in the range of 8 Å to 10 Å, and the long-range correction applied to account for the effect of Lennard-Jones interactions beyond the truncation [36]. The nonbonded pairlist contained atoms pairs separated by up to 12 Å and was updated whenever any atom's displacement relative to its position at the last pairlist update exceeded 1 Å. Finally, each protein+water+ion system was minimized for 5000 steps with steepest-descent followed by 5000 steps of conjugate gradient prior to the MD calculations.

### Molecular dynamics

Each of the three systems – 101,024 atoms and a minimum dimension of 112.5 Å for the full protein; 25,244 atoms and a minimum dimension of 71.1 Å for N-SH2 from the full protein crystal structure; and 22,031 atoms and a minimum dimension of 68.1 Å for N-SH2 from the N-SH2-only crystal structure – was simulated in the isothermal-isobaric ensemble. The system was propagated using the 'leap-frog' algorithm to integrate the equations of motion [39]. Temperature was maintained at 298 K by a Nosé-Hoover heat bath [40,41] with a thermal piston parameter of 10,000 kcal*mol^-1^*ps^2^, and pressure was maintained at 1 atm using the Langevin piston [42] with a piston mass of 1000 amu, a collision frequency of 10 ps^-1^, and coupled to a temperature bath of 298 K. Each 5-ns or 10-ns simulation was preceded by a 20-ps heating interval in which the Nosé-Hoover heat bath was replaced by rescaling of atomic velocities every 0.1 ps and in which the Langevin piston collision frequency was 25 ps^-1^.

### φ/ψ free-energy surface calculations

Potential of mean force (PMF) calculations were undertaken to calculate conformational free energies of the Tyr66 in the left-handed helical and extended regions of φ/ψ space. Rectangular portions of both regions were sampled, with the left-handed helical conformations having φ values ranging from 30° to 110° and ψ values from 0° to 80°, and extended conformations having φ values ranging from -110° to -20° and ψ values from 70° to 150°. To bias sampling of Tyr66 to a particular region of φ/ψ space, harmonic restraining potentials of the form *E *= *k*·(*θ *- *θ*_min_)^2 ^were applied to the φ and ψ dihedrals. The value of *k *was incremented over the course of 20 ps: *k *was set to 0.003 kcal*mol^-1^*degree^-2 ^for 5 ps, then increased to 0.005 for another 5 ps, then to 0.010 for another 5 ps, and then to 0.025 for a final 5 ps. The system was then simulated for 200 ps with *k *= 0.025 kcal*mol^-1^*degree^-2^. For the left-handed alpha helical region, φ_min_/ψ_min _were initially set to 70°/40° and the starting conformation was that of 1AYD:N-SH2 at 10 ns, whose Tyr66 φ/ψ values were 74°/37°. The final snapshot from this biased simulation was used as the starting point for four other simulations with φ_min_/ψ_min _values of 60°/40°, 80°/40°, 70°/30° and 70°/50° and using the same scheme that started with *k *= 0.003 kcal*mol^-1^*degree^-2^, incremented it to 0.025 kcal*mol^-1^*degree^-2 ^over 20 ps, and followed by a 200 ps trajectory. Subsequent simulations similarly employed the final conformation from prior simulations that had sampled a directly adjacent region in φ/ψ space, φ_min_/ψ_min _were always a multiple of 10 degrees, and the same *k *incrementing scheme was applied. For the extended region, φ_min_/ψ_min _were initially set to -60°/100° and the starting conformation was that of 2SHP:N-SH2 at 10 ns, whose Tyr66 φ/ψ values were -64°/98°. The molecular dynamics methods (heat/pressure bath, integrator, long-range truncation, etc.) were otherwise the same as for the unrestrained molecular dynamics simulations.

Tyr66 φ/ψ values were saved every dynamics step during the 200-ps *k *= 0.025 kcal*mol^-1^*degree^-2 ^phase. Using the weighted histogram analysis method [43,44], the φ/ψ probability distributions from the simulations were properly reweighted and combined to generate free-energy surfaces for the 20–80 ps, 80–140 ps, and 140–200 ps intervals. The resulting free-energy surfaces were invariant with time, thus the combined data from the 20–200 ps intervals was used in the analysis.

### Induction of Tyr66 backbone conformational change

The same φ/ψ restraining potentials as used in the free-energy surface calculations were applied to induce changes in the Tyr66 backbone conformation. Starting conformations were the final snapshots from the 10-ns 1AYD:N-SH2 and 2SHP:N-SH2 trajectories. The change in φ_min_/ψ_min _followed the path described in the Results and Discussion and each subsequent simulation following alteration of either φ_min _or ψ_min _changed by 10° used the final snapshot from the previous simulation. *k *assumed the same values as in the free-energy simulations, but was incremented every 0.5 ps, instead of every 5 ps and was followed by a 20-ps trajectory instead of a 200-ps trajectory. 44 values of φ_min_/ψ_min _were required to span the reaction path, thus the conformational change was induced over a total of 44 * 22 ps = 968 ps. The MD protocols were the same as for the unrestrained molecular dynamics simulations.

## List of abbreviations

SH2, SRC homology 2 domain

PTP, protein tyrosine phosphatase domain

N-SH2, N-terminal SH2 domain

C-SH2, C-terminal SH2 domain

pY, phosphotyrosine

MD, molecular dynamics

PMF, potential-of-mean-force

1AYD:N-SH2, N-SH2 system constructed from [PDB:1AYD] x-ray crystal coordinates

2SHP:N-SH2, N-SH2 system constructed from [PDB:2SHP] x-ray crystal coordinates

2SHP:SHP-2, SHP-2 system constructed from [PDB:2SHP] x-ray crystal coordinates

RMSD, root mean square deviation

E, extended region of φ/ψ space

H, helical region of φ/ψ space

L, left-handed helical region of φ/ψ space

G, gamma region of φ/ψ space

2, II' region of φ/ψ space

## Authors' contributions

OG set up the calculations, performed all analysis and prepared the manuscript with input from CKQ and ADM. ADM and CKQ contributed to the design of the calculations and interpretation of the data and ADM coordinated the study. All authors read and approved the final manuscript.

## Figures and Tables

**Table 1:** Summary of the three simulated systems

***System description***	***Crystal structure used for construction***	***Residue span***	***pY-peptide binding cleft state in crystal***^†^	***Abbreviation for system***
Isolated N-SH2	Isolated N-SH2 [PDB:1AYD]	1–108	Open	1AYD:N-SH2
Isolated N-SH2	Full SHP-2 [PDB:2SHP]	1–108	Closed	2SHP:N-SH2
Full SHP-2	Full SHP-2 [PDB:2SHP]	1–525	Closed	2SHP:SHP-2

^† ^Crystals did not have bound pY-peptide.
